# Stent Graft Restenosis: Insights From Optical Coherence Tomography Imaging and Treatment Considerations

**DOI:** 10.7759/cureus.72696

**Published:** 2024-10-30

**Authors:** Hiroshi Abe, Dai Ozaki, Hiroyuki Isogai, Takashi Tokano, Tohru Minamino

**Affiliations:** 1 Cardiology, Juntendo University Urayasu Hospital, Urayasu, JPN; 2 Cardiovascular Biology and Medicine, Juntendo University Graduate School of Medicine, Hongouku, JPN

**Keywords:** endovascular stent graft, evt: endovascular therapy, instent restenosis, in-stent restenosis- mechanism- history- management, intravascular ultrasound (ivus), oct (optical coherence tomography)

## Abstract

Endovascular therapy (EVT) utilizing self-expanding stent grafts has shown promising clinical outcomes for femoropopliteal lesions. However, restenosis and thrombotic occlusion remain significant concerns with unclear underlying mechanisms. This is the case of a 53-year-old male with diabetic nephropathy requiring hemodialysis. He presented with left lower limb intermittent claudication and underwent EVT, resulting in subsequent restenosis 12 months post-implantation and recurrence of symptoms. Intravascular imaging revealed neointimal proliferation and stent-edge restenosis, with segments of neointima not in contact with the stent. A second EVT procedure was performed successfully with the endograft extension. Our case highlights the challenges in diagnosing and managing stent graft restenosis. Intravascular imaging modalities such as optical coherence tomography (OCT) were crucial in identifying the etiology and guiding treatment decisions. The presence of neointima not in contact with the stent suggests a potential mechanism for thrombus formation and underscores the importance of tailored treatment strategies. This case underscores the utility of OCT in evaluating stent graft restenosis and guiding therapeutic interventions. Further research is warranted to elucidate optimal treatment strategies for this challenging complication.

## Introduction

Peripheral arterial disease of the lower extremities (LEAD) is marked by reduced blood flow and insufficient oxygen supply to the lower limbs, caused by the narrowing and obstruction of the arteries. Endovascular therapy (EVT) should be the first choice for treating femoropopliteal lesions, even for complex lesions, especially in patients at high surgical risk [1]. EVT faces ongoing challenges in maintaining long-term patency and stability, particularly in the highly mobile femoropopliteal artery following stent placement. Drug-eluting balloons have demonstrated improved outcomes in more complex cases [2]. However, concerns over paclitaxel-coated devices emerged after a meta-analysis prompted a decrease in their use [3]. Subsequent evaluations of large-scale data failed to confirm the increased mortality risk, leading the FDA to reassess [4]. Currently, drug-eluting devices are considered both safe and effective for treating femoropopliteal lesions. EVT using self-expanding stent grafts has shown satisfactory clinical outcomes in treating femoropopliteal lesions [5,6]. While restenosis and thrombotic occlusion are known, the detailed mechanisms remain largely unclear.

## Case presentation

**Figure 1:**
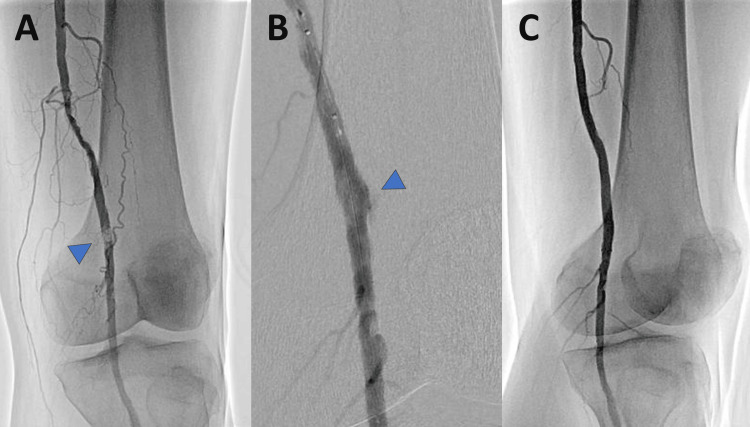
The initial EVT procedure. (A) Angiography revealed severe stenosis with calcified plaque in the left superficial femoral artery to the popliteal artery. (B) Balloon dilation resulted in vascular perforation. (C) Successful expansion and imaging findings were obtained following the replacement of a 6.0 mm × 100 mm heparin-bonded, self-expanding stent graft. EVT, endovascular therapy

This is the case of a 53-year-old male patient who underwent hemodialysis at the age of 44 due to diabetic nephropathy. The patient had concomitant conditions of diabetes, dyslipidemia, and hypertension and was referred to our institution due to intermittent claudication in the left lower limb. An ankle-brachial index (ABI) of 0.74 was observed, and lower limb angiography revealed severe stenosis with calcified plaque in the left superficial femoral artery to the popliteal artery. Using the Arcadia technique [7] to traverse the calcified plaque with a wire and balloon dilation resulted in vascular perforation, necessitating the placement of a 6.0 mm × 100 mm heparin-bonded, self-expanding stent graft (Viabahn; W.L. Gore & Associates, Flagstaff, AZ; Figure 1).

**Figure 2:**
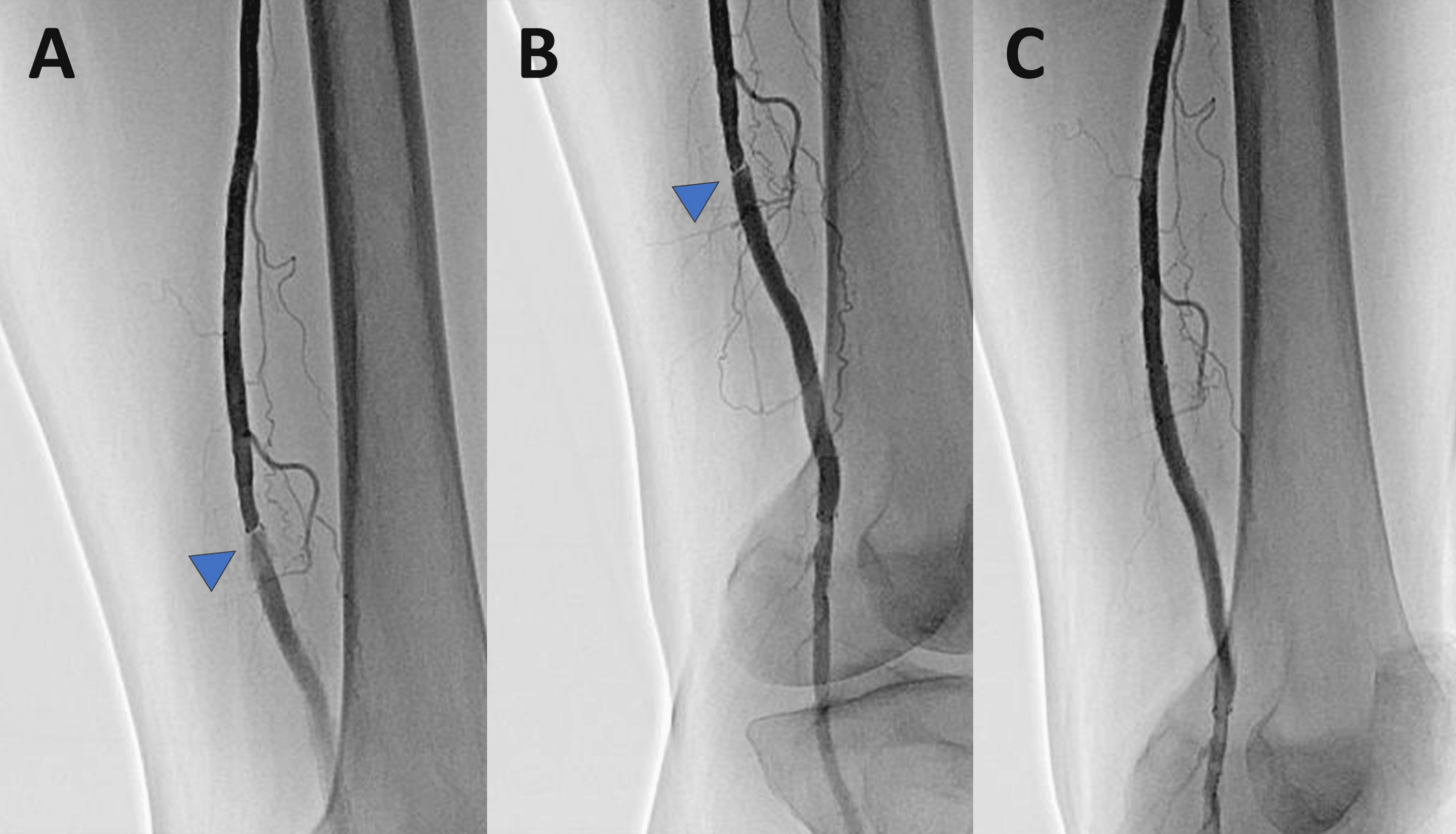
Angiography and EVT procedure following in-stent restenosis. (A) Angiography revealed severe stenosis. (B) Retrograde flow staining within the stent could be observed post-stenosis. (C) Successful expansion and imaging findings were obtained following the replacement of a 6.0 × 50 mm heparin-bonded, self-expanding stent graft. EVT, endovascular therapy

The ABI improved to 1.19, and intermittent claudication showed improvement. The patient was on aspirin (100 mg/day) and clopidogrel (75 mg/day) therapy. Twelve months post-implantation, the ABI decreased to 0.76, and vascular ultrasound revealed suspected restenosis at the proximal end of the stent. Concurrently, a recurrence of intermittent claudication was noted. Despite continued exercise therapy, no improvement in intermittent claudication was observed, leading to EVT 23 months post-implantation. Lower limb angiography revealed contrast-filling defects above the stent at the proximal end (Figures 2A-2B).

**Figure 3:**
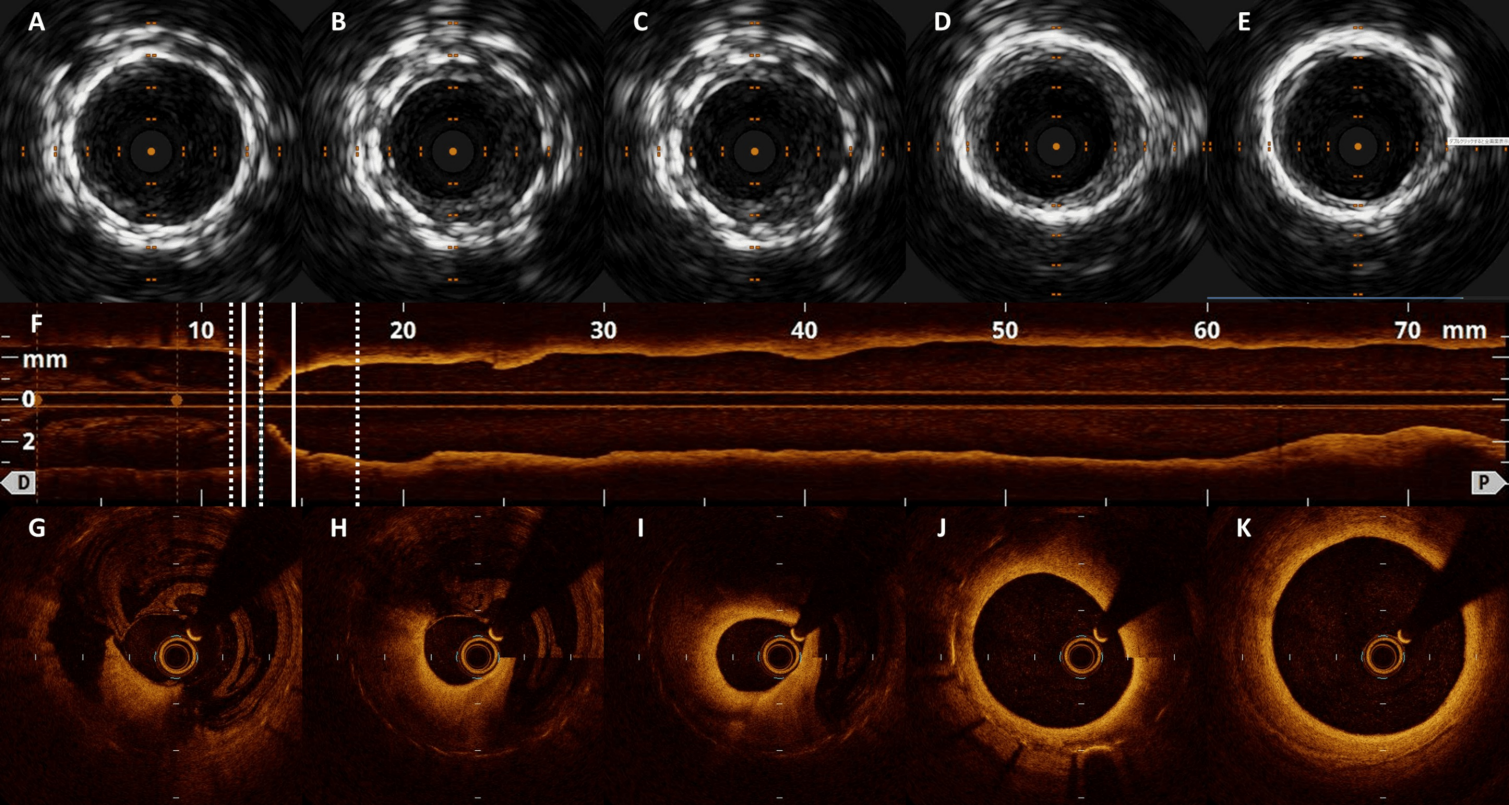
The images of IVUS and OCT. The images show observations from distal to proximal in the order of A-E, G-K. Corresponding positions in OCT image F are shown for A and G, B and H (A-E) IVUS identified thin, low-signal neointimal hyperplasia with luminal area of 9.1 mm^2^. (F-K) OCT showed neointimal proliferation, resulting in significant stenosis with a luminal area of 1.59 mm^2^. Approximately 180° was not in contact with the vessel wall. IVUS, intravascular ultrasound; OCT, optical coherence tomography

Intravascular ultrasound (IVUS) identified thin, low-signal neointimal hyperplasia within the stent but with a preserved luminal area of 9.1 mm^2^. Optical coherence tomography (OCT) showed continuous neointimal proliferation from the proximal end of the stent, resulting in significant stenosis with a luminal area of 1.59 mm^2^. Approximately 180° of the stent near the proximal end was not in contact with the vessel wall and was observed floating within the vessel, resulting in stagnant blood flow in this region (Figure 3).

A heparin-bonded, self-expanding 6.0 mm × 50 mm stent graft (Viabahn; W.L. Gore & Associates, Flagstaff, AZ) was deployed to cover the neointima, concluding the EVT (Figure 2C). The ABI improved from 0.76 to 1.18, with the resolution of intermittent claudication.

## Discussion

This case demonstrated stent-edge restenosis accompanied by neointimal proliferation, with OCT revealing segments of the neointima not in contact with the stent floating within the vessel.

Lammer et al. reported a one-year primary patency of stent graft implantation of 78.1%, with all nine cases reporting stent-edge restenosis [5]. In the VIPER (Viabahn Endoprosthesis with Heparin Bioactive Surface in the Treatment of Superficial Femoral Artery Obstructive Disease) trial, 17 out of 18 cases reported stent-edge restenosis at one year [6]. Ishihara et al. reported neointimal hyperplasia at the stent edge histologically in a post-mortem examination 23 months after stent graft placement [8].

In addition to the previous reports, neointimal proliferation in the stent graft is expected to continue to grow parallelly from the stent edge. This scenario underscores the importance of proper management and follow-up, necessitating measures to minimize the risk of restenosis. While IVUS provided limited clarity, OCT enabled detailed observation of the vessel status of stent restenosis. IVUS, using a transducer to collect information by reflecting ultrasound from within the vessel, may have difficulty in clear visualization when the intima is not in contact with the vessel wall, as seen in this case. OCT allows for detailed structural observation compared to IVUS but has a shallower depth and is less commonly used for lower limb arteries due to larger vessel diameters and challenges in blood clearance. Successful OCT imaging was achieved by an ipsilateral femoral artery approach and compression of the sheath insertion site to minimize blood inflow. Discrepancies between clinical findings, lower limb angiography, and IVUS findings highlight the importance of OCT in determining treatment strategies. Even in cases where IVUS evaluation reveals discrepancies with clinical findings, innovative use of OCT can facilitate accurate lesion assessment and treatment strategy determination.

Segments of neointima not in contact with the stent may create pockets of stagnant blood flow, as observed on OCT and lower limb angiography. Stent graft thrombosis has been reported, with speculation that the presence of neointima not covered by stent grafts may promote thrombus formation by initiating coagulation reactions via tissue factors in the noncovered stent midsection [9]. In cases of stent-edge thrombosis, as in this case, where segments of neointima are not in contact with the stent, the possibility of thrombus formation from two of Virchow's triad [10], hypercoagulability of blood and alteration in blood flow in the vessels, is considered. In cases where neointima is not in contact with the stent edge and stent-edge restenosis is observed, treatment with drug-coated balloons (DCBs) or balloon angioplasty may not be effective due to lack of neointimal contact, making stent-based therapy a potentially effective treatment option.

Effective treatment strategies for stent graft restenosis are not currently established, highlighting the importance of elucidating etiology and establishing treatment strategies tailored to the etiology, necessitating future investigation.

## Conclusions

In conclusion, this case underscores the challenges of managing stent graft restenosis, particularly highlighting the utility of OCT in identifying critical morphological features of restenosis. The discovery that segments of the neointima were not in contact with the stent was pivotal, influencing further intervention strategies. This instance emphasizes the necessity for selecting precise imaging modalities that can uncover essential details, thereby guiding complex vascular treatment strategies. Furthermore, it underscores the need for ongoing research into neointimal proliferation and stent graft restenosis mechanisms to develop more targeted and effective interventions for such conditions.
